# The Influence of the COVID-19 Pandemic on Seasonal Influenza Vaccine Uptake Among Patients Visiting a University Hospital in Saudi Arabia

**DOI:** 10.7759/cureus.47042

**Published:** 2023-10-14

**Authors:** Shabana Tharkar, Shatha Alduraywish, Abdul Aziz Nishat, Lamis Alsuwailem, Lina Alohali, Mashael K Kahtani, Fahad M Aldakheel

**Affiliations:** 1 Prince Sattam bin Abdulaziz Research Chair for Epidemiology and Public Health, Department of Family and Community Medicine, College of Medicine, King Saud University, Riyadh, SAU; 2 Department of Family and Community Medicine, College of Medicine, King Saud University, Riyadh, SAU; 3 Department of Biomedical Science, School of Biomedical, Nutritional and Sports Sciences, Newcastle University, Newcastle upon Tyne, GBR; 4 College of Medicine, King Saud University, Riyadh, SAU; 5 Department of Clinical Laboratory Science, College of Applied Medical Science, King Saud University, Riyadh, SAU

**Keywords:** seasonal influenza, saudi arabia, influenza vaccination hesitancy, influenza vaccination acceptance, covid-19 pandemic

## Abstract

Introduction: Influenza vaccination is a subject of importance in Saudi Arabia. The study measured the uptake of annual influenza vaccination from 2019 to 2021 among patients attending outpatient clinic of a University Hospital.

Materials and methods: A cross-sectional study design was used, and the questionnaire was administered by trained interviewers. Descriptive and inferential statistics were done using the Statistical Package for the Social Sciences (SPSS) version 21 (IBM, Armonk, New York).

Results: The three-year annual influenza vaccine uptake for 2019-2021 was 19.7%, 11.4%, and 14.2%, respectively. In the year 2022, only 28.2% of the patients were offered influenza vaccines by their physicians, and among those offered, 49.6% showed vaccine acceptance. Higher vaccine acceptance was significantly associated with past episodes of influenza infection (p<0.001) and vaccination history before the COVID-19 pandemic (p<0.001). Lower acceptance of the influenza vaccine was observed during the pandemic (p<0.001) and lower uptake among those who were not offered influenza vaccines (p=0.02). No association was found between influenza vaccine acceptance and smoking status, chronic illness, history of COVID-19 infection, or living with those susceptible to influenza. Reasons for vaccine denial include an assumption of not being at risk, a lack of information about the vaccine, and a fear of side effects.

Conclusion: The COVID-19 pandemic has had a detrimental effect on annual influenza vaccination. Efforts must be taken to increase influenza vaccination among vulnerable groups.

## Introduction

Seasonal influenza, a viral infection of the respiratory tract that may lead to pneumonia or respiratory failure, is the fifth leading infectious disease and among the top ten causes of mortality worldwide, causing an enormous public health burden [1]. The estimates of the burden of illness reported by the Global Burden of Disease (GBD) study in 2017, stand at 9,459,000 hospitalizations, 145,000 (99,000-200,000) deaths among all ages, and 54,481,000 episodes attributed to influenza [1]. The annual seasonal influenza infection is characterized by cough, runny nose, muscle aches, fatigue, and fever, and sometimes reaches epidemic proportions during the winter months [2]. More severe illness is reported in vulnerable populations like the elderly (70 years and older), people with multiple comorbidities, infants and children, and pregnant women. The causative organism Influenza virus A and B types have further classifications based on serological and genetic typing that constantly undergo mutations leading to the emergence of new variants by antigenic drifts [3]. The most effective way of preventing illness is through vaccination, as recommended by various health organizations. The World Health Organization (WHO) recommends annual influenza vaccination for the identified risk groups, which are children under twelve years, pregnant women, people older than 65 years, those with chronic disease conditions, and health workers [4]. The Centers for Disease Control and Prevention (CDC) estimates that annual influenza vaccination prevents at least 4.4 million cases and 3500 deaths in the United States annually [5].

The reported annual influenza incidence in Saudi Arabia is 773.8 per 100,000 population with an average of 267,000 annual episodes of influenza infection in the population. The estimated annual hospitalization rate and mortality rate attributed to influenza are 36,000 (12000 to 104000) and 0.8 deaths per 100,000 population, respectively [1]. Saudi Arabia has a relatively hot desert climate, and winter typically lasts between October and March in the central and northern regions with temperatures ranging between 10 and 17 °C during the day and 0-10 °C at night. Influenza epidemics have a typical seasonal existence with the primary peak occurring in winter (November to February) and a smaller secondary peak during the spring season. In addition, mass gathering events like the holy Hajj pilgrimage in the cities of Makkah and Madinah pose an additional risk of transmission of infections [6].

The ongoing SARS-CoV-2 pandemic (also known as the COVID-19 pandemic) has affected over 690.2 million people and had caused more than 6.9 million deaths globally by June 2023 [7]. The causative agent is a novel coronavirus with yet unclear etiology first detected in the Wuhan province of China in December 2019 [8]. Both influenza and COVID-19 infections share common disease symptoms and transmission. Both have viral origins, affecting the respiratory tract predominantly, causing symptoms like fever, cough, and myalgia. They are transmitted by respiratory droplets and can be prevented to an extent by vaccines.

Due to the high rates of human-to-human transmission of the SARS-CoV-2 infection, WHO declared it a pandemic as early as March 2020. By 2021, the virus had undergone several mutations, and the CDC classified ten variants as variants being monitored (VBM) and two major variants of concern (VOC) that were labeled as Delta and Omicron, which wreaked havoc, especially during the winter [9]. Progressively, subvariants of Omicron like BA.1, BA.2, and BA.5 began to emerge in early 2022, which were declared highly transmissible but with less severity of infection compared to their predecessors and were declared variants of concern [10]. However, in late 2022, another Omicron subvariant and lineage of concern BQ.1.1 emerged causing a large proportion of infections [10].

With the introduction of coronavirus vaccines as early as December 2020, vaccination against the COVID-19 infection stepped up across the globe, with some countries mandating first and second doses or even the booster or third dose of the anti-coronavirus vaccine. Saudi Arabia is among the first few countries that started COVID-19 vaccination in December 2020. Active cases of COVID-19 from January to June 2023 have been within the range of 3000-5000 cases, while daily new cases have shown to slightly increase since April 2023, with the highest reported peak on 11th April reaching 401 cases. Even though the trend shows a fluctuating pattern, caution must be exercised to prevent higher peaks in the later months of 2023 due to the constant risk from the high volume of mass gatherings related to pilgrimage. 

Simultaneously, greater emphasis has also been made on influenza vaccination and the Ministry of Health (MOH) in Saudi Arabia, released a health advisory that all people get vaccinated against influenza to prevent influenza infection during the ongoing COVID-19 pandemic, especially during the winter months between October and March [11]. Research on influenza vaccine prevalence and acceptance during the ongoing pandemic in Saudi Arabia is sparse, and only a few studies have reported the findings. Alkathlan et al. reported a decreased uptake of influenza vaccination during the pandemic compared to pre-pandemic times among healthcare professionals [12]. Another study reported a 19.3% uptake of influenza vaccination among confirmed COVID-19 patients [13]. Pre-pandemic influenza vaccination data among older adults showed a 47% vaccination rate at least once [14]. However, not much data exists on general patients, who may be categorized as a high-risk group for influenza vaccination. Hence, this study investigated the influenza vaccine uptake among the patient population during the COVID-19 pandemic and identified the determinants of vaccine uptake among patients attending an outpatient clinic at a University Hospital. The study findings may provide valuable information to health policymakers and health professionals for future pandemic preparedness.

## Materials and methods

Study design and participants

An observational cross-sectional study design was used to measure the three years (2019, 2020, and 2021) uptake of influenza vaccination, vaccine acceptance/denial in 2022, knowledge of influenza symptoms and transmission, knowledge about influenza vaccines and the effect of COVID-19 pandemic on influenza vaccine acceptance and denial. The study participants included patients aged greater than 18 years attending the outpatient clinic of a large tertiary care University Hospital. The hospital is one of the largest public sector organizations catering to the needs of the population, providing free healthcare from primary to super specialty levels of care. Patients who were incapable of comprehending the questions, febrile patients who were unable to answer the questions, and those who refused to participate were not included in the study.

Questionnaire and study variables

The questionnaire was adopted from a previous study that measured influenza vaccine uptake among medical students [15]. However, modifications were made suitably by adding questions related to the COVID-19 pandemic and modifying the target population, which included the patients. The questionnaire contained 17 questions in two sections. The first section assessed information on socio-demographic variables, COVID-19 history, and chronic disease status. The second section collected information about knowledge of seasonal influenza vaccination, past immunization practices, vaccine acceptance and hesitancy. The study intended to measure the influenza vaccine uptake, which forms the main study variable. Independent variables include socio-demographic characteristics, smoking status, chronic disease status, seasonal flu history and past immunization status, and COVID-19 history and hospitalization details. Dependent variables include influenza vaccine acceptance and hesitancy.

Vaccine uptake refers to having been vaccinated, vaccine acceptance refers to the degree to which people will accept the vaccine when offered, and vaccine denial refers to not accepting the vaccine when offered. 

Sample size and data collection

The sample size was calculated using the single proportion formula: n=Z2pq/d2, where Z is the 95% confidence interval, which equals to 1.96, and ‘d’ is the precision of 5%. P is the prevalence of vaccination in a different study population, and q is equal to (1-p). Considering ‘p’ as 21% of the vaccination rate among the medical students from a previous study [15], the sample size was estimated at 254.

Three researchers had undergone intensive training in data collection by a senior scientist who was involved in designing the questionnaire. The training was conducted in three theoretical and two practical sessions. The administrators exchanged the interview process with each other and later extended the data collection to their colleagues as part of practical training. 

Since questions related to the COVID-19 pandemic were inserted in the standardized questionnaire, a pilot study was performed on twenty patients to test the validity of the newly added questions. This data was not included in the main study. The final questionnaire was then made ready for data collection after review by the expert committee. The questionnaire was entered in Google Docs, and an electronic version was made. The e-questionnaire was then administered by the trained personnel to the study patients. Patients greater than 18 years of age attending the outpatient clinic were randomly selected and enrolled in the study after obtaining informed consent.

Statistical analysis

All the data analysis was done using Statistical Package for the Social Sciences (SPSS), version 21 (IBM, Armonk, New York). Descriptive statistics included reporting data in frequencies and percentages or mean and standard deviation as appropriate for categorical and continuous variables. Inferential statistics included the chi-square test of proportions and an ANOVA. A p-value of less than 0.05 was considered significant.

Ethical considerations

The participant's anonymity was maintained, and none of the questions disclosed identity. Informed consent was obtained from every study subject prior to the start of the survey. Study approval was obtained from the Institution's Review Board Committee with Ref. No. 21/0932/IRB.

## Results

Socio-demographic characteristics of the study participants

A total of 254 subjects (117 males and 135 females) participated in the study. Around 56.7% of the study population were below 40 years old. Among them, 34 (13.4%) were identified as current smokers, and 28 (11%) were ex-smokers. Higher rates of smoking were found in men. Chronic disease status, influenza, and vaccination history are depicted gender-wise in Table 1.

**Table 1:** General demographic characteristics of the study population. IBD: irritable bowel disease, HCP: health care professionals.

Variables		Male 117 (46.1%)	Female 137 (53.9%)	P-value
Age (years)	20-29	34 (29.1)	33 (24.1)	-
	30-39	29 (24.8)	48 (35.0)	
	40-49	26 (22.2)	25 (18.2)	
	>50	28 (23.9)	31 (22.6)	
Smoking status	Never smoker	61 (52.1)	131 (95.6)	0.001
	Ever smoker	56 (47.9)	6 (4.4)	
Chronic illness	Present	51 (43.6)	57 (41.6)	0.42
	Absent	66 (56.4)	80 (58.4)	
Types of common chronic diseases	Asthma	9 (7.7)	21 (15.3)	0.04
	Diabetes	28 (23.9)	23 (16.8)	0.10
	Hypertension	27 (23.1)	28 (20.4)	0.36
	IBD	9 (7.7)	13 (9.5)	0.39
Living with susceptible individuals	Under 16 years	77 (65.8)	80 (58.4)	0.13
	Pregnant women	8 (6.8)	19 (14)	0.05
	Over 65 years	39 (33.3)	52 (38)	0.26
	HCP	26 (22.2)	38 (27.7)	0.19
History of seasonal flu	During pandemic	51 (43.6)	76 (55.5)	0.03
	Before pandemic	84 (71.8)	108 (78.8)	0.12
History of COVID-19 infection		38(32.5)	56 (40.9)	0.10
Seasonal flu vaccine history	2021	18 (15.4)	18 (13.1)	0.36
	2020	13 (11.1)	16 (11.6)	0.52
	2019	21 (18)	29 (21.2)	0.31
	Before 2019	29 (24.8)	33 (24.1)	0.50
Vaccine offered	Yes	32 (27.3)	40 (29.2)	0.42
Vaccine acceptance if offered	Yes	58 (49.6)	71 (51.8)	0.40

Knowledge of influenza infection and vaccine

Influenza vaccination priority groups and the mode of transmission of the seasonal flu virus were assessed and displayed in Table 2. The mean knowledge scores were significantly higher for women (male: 6.4±1.3; female: 7±1.3; p=0.01). The minimum and maximum scores were 3 and 9. Approximately 92 (78.6%) men and 119 (86.9%) women achieved a mean score of 6 and above. The answers to both questions on priority groups for vaccination and transmission of the virus were similar in distribution among men and women. Being pregnant was prioritized by women as a high priority for receiving influenza vaccination, which showed a significant difference with the opinion of men (34:29.1%; 63:46%; p=0.004). 

**Table 2:** Knowledge of influenza infection and vaccine priority groups.

Knowledge questions		Male 117 (46.1%) n(%)	Female 137 (53.9%) n(%)	P-value
Priority group for receiving seasonal influenza vaccine	Over 65	88 (75.2)	112 (81.8)	0.13
	Under 16	52 (44.4)	56 (40.9)	0.32
	Pregnant women	34 (29.1)	63 (46)	0.004
	Healthcare professionals at home	103 (88)	124 (90.5)	0.33
	People with chronic health conditions	96 (82.1)	121 (88.3)	0.10
Methods of transmission of seasonal influenza virus	Cough/sneeze	113 (96.6)	133 (97.1)	0.54
	Eating infected meat	28 (23.9)	22 (16.1)	0.07
	Direct contact with an infected person	106 (90.6)	127 (92.7)	0.35
	Touching contaminated object	79 (67.5)	90 (65.7)	0.43
Total knowledge score	Mean (SD)	6.4±1.3	7±1.3	0.01

Past years vaccine uptake: To the question about past years vaccine uptake, in 2019, 2020, and 2021, 19.7%, 11.4%, and 14.2% responded positively for having taken the vaccine (data not shown in table).

Determinants of vaccine acceptance in 2022

The determinants of vaccine acceptance among those who were offered vaccines by their physicians are shown in Table 3. Of the total study population, only 72(28.3%) of the participants were offered vaccines by their physicians. The influenza vaccine was similarly offered across gender and smoking status or the presence of chronic illness and living with susceptible individuals did not make any difference in the prescribing of the influenza vaccine. However, a past seasonal influenza vaccination history among men (p=0.01) and having not taken influenza vaccination during the COVID-19 pandemic (p<0.001) were significantly higher in receiving the vaccination offer. 

**Table 3:** Determinants of vaccine acceptance among those offered vaccine.

Variable		Vaccine offered 72(28.3) n(%)	Vaccine not offered 182 (71.7) n(%)	P-value
Gender	Male	32 (44.4)	85 (46.7)	0.42
	Female	40 (55.6)	97 (53.3)	
Smoking status	Ever smoker	20 (27.8)	42 (23.1)	0.26
	Never smoker	52 (72.2)	140 (76.9)	
Chronic illness	Yes	44 (61.1)	102 (56)	0.27
	No	28 (38.9)	80 (44)	
Living with susceptible individuals	Yes	61 (84.7)	153 (84.1)	0.53
	No	11 (15.3)	29 (15.9)	
Seasonal influenza past vaccination history	Yes	39 (54.2)	68 (37.4)	0.01
	No	33 (45.8)	114 (62.8)	
Seasonal influenza vaccination during COVID-19 pandemic	Yes	34 (47.2)	15 (8.2)	0.001
	No	38 (52.8)	167 (91.8)	

The determinants of vaccine acceptance are tabulated in Table 4. Of the total study participants, 161 (63.4%) accepted to take the influenza vaccine if offered. Both acceptance and denial of the influenza vaccine were remarkably similar across the gender. Similarly, the current smoking status, having a chronic illness, and living with susceptible persons did not show any significant difference in influenza vaccine uptake. However, a positive past history of seasonal influenza (p<0.001), those who have not been vaccinated against the influenza virus during the COVID-19 pandemic (p<0.001), and those who had been taking the influenza vaccine before the COVID-19 pandemic (p<0.001) and among those who received an offer of influenza vaccine (p=0.02) showed increased vaccine acceptance. Vaccine denial was also expressed among those who had been denying the influenza vaccine in the past years.

**Table 4:** Determinants of vaccine acceptance and denial among the whole study population.

Variable		Vaccine acceptance 161 (63.4) n(%)	Vaccine denial 93 (36.6) n(%)	P-value
Gender	Male	74 (46)	43 (46.2)	0.53
	Female	87 (54)	50 (53.8)	
Age	20-29	36 (53.7)	31 (46.3)	0.10
	30-39	43 (55.8)	34 (44.2)	
	40-49	18 (35.3)	33 (64.7)	
	50-59	32 (54.2)	27 (45.8)	
Smoking status	Ever smoker	38 (23.6)	24 (25.8)	0.40
	Never smoker	123 (76.4)	69 (74.2)	
Chronic illness	Yes	93 (57.8)	53 (57)	0.50
	No	68 (42.2)	40 (43)	
Living with susceptible individuals	Yes	136 (84.5)	78 (83.9)	0.51
	No	25 (15.5)	15 (16.1)	
Past seasonal influenza infection history	Yes	91 (56.5)	36 (38.7)	0.001
	No	70 (43.5)	57 (61.3)	
Seasonal influenza vaccination during COVID-19 pandemic	Yes	46 (28.6)	3 (3.2)	0.001
	No	115 (71.4)	90 (96.8)	
Seasonal influenza vaccination before COVID-19 pandemic	Yes	87 (54)	20 (21.5)	0.001
	No	74 (46)	73 (78.5)	
History of COVID-19 infection	Yes	42 (26)	52(56)	0.08
	No	87 (54)	73 (78.5)	
Influenza vaccine offered	Yes	53 (33)	19 (20.4)	0.02
	No	108 (67)	74 (79.6)	

**Figure 1:**
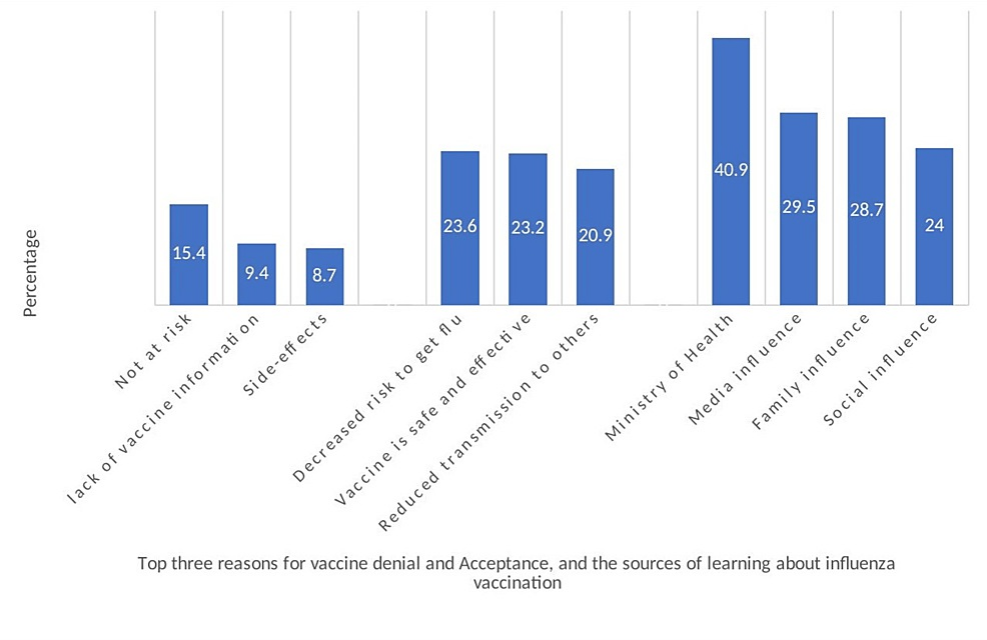
Illustration of the most commonly cited reasons for vaccine denial and acceptance and sources of information. Data is reported in percentage.

The most common reasons for vaccine denial, acceptance, and sources of information-seeking behavior are illustrated in Figure 1. An expression of not being at risk of acquiring influenza (15.4%) was the most common reason cited for vaccine denial. Decreased risk of disease transmission (23.5%), safety, and efficacy of vaccine (23.2%) were the most commonly cited reasons for vaccine acceptance. The Ministry of Health (40.9%) was the single most common source of vaccine-related health-seeking behavior, followed by media (29.5%) and family (28.7%) influence.

## Discussion

Saudi Arabia achieved one of the highest rates of coverage of the COVID-19 vaccination and is one among very few countries that mandated third booster doses [16,17]. The MOH also emphasized seasonal influenza vaccination to the susceptible individuals and recommended considering immunizing with the influenza vaccine in addition to the full doses of the COVID-19 vaccine to the high-risk groups to prevent influenza sickness amidst the coronavirus pandemic. The annual seasonal influenza vaccination in Saudi Arabia had previously remained sub-optimal, and the MOH in 2015 increased the targeted coverage to 30% for the high-risk groups [18]. The impact of the COVID-19 pandemic and its vaccination on annual influenza vaccination remains largely unexplored in the high-risk categories in Saudi Arabia. Hence, the present study investigated the last three-year trend of the seasonal influenza vaccination rate, the uptake in the current year, and the reasons for acceptability and denial among the patients attending a tertiary hospital.

With the relaxation of restrictions, co-infections from other viral infections and influenza sickness may pose an additional burden. Hence, the WHO also recommended prioritizing influenza vaccination to the vulnerable and high-risk population during the pandemic since there has been a continuous threat from the ever-evolving subvariants [19]. 

The three-year trend of influenza vaccination uptake

From the present study, the uptake of annual influenza vaccination among the study patients in 2019, 2020, and 2021 was 19.7%, 11.4%, and 14.2%, respectively. The cumulative for the last three-year period was 45.3%. The vaccine uptake was almost similar across the gender. The study found a slightly higher influenza vaccine uptake before the pandemic and then showed a decline to 11.4% in the first year of the pandemic. In other words, influenza vaccine hesitancy increased during the first year of the COVID-19 pandemic.

Pre-pandemic studies from the region of Saudi Arabia have been reporting sub-optimal uptake of seasonal influenza vaccination among the general population, healthcare workers, and high-risk groups [14,15,20,21]. Nevertheless, the seasonal flu vaccination trend during the COVID-19 pandemic has aggravated the decline in uptake. In comparison, a previous study by AbalKhail et al. reported a three-year cumulative trend of 57% for pre-pandemic influenza immunization, and the three-year trend declined by 12% among medical students, who are one of the high-risk categories [15]. However, reports from western nations are in stark contradiction with our findings. Data from the United Kingdom showed higher influenza vaccine uptake among general patients visiting the general practice (GP). The uptake was almost 37% among pregnant women, 82% in those greater than 65 years, and 45% among those between 45 and 64 years [22]. One of the main possible reasons could be related to the higher prevalence of coronavirus cases and the increased burden of illness in Europe compared to Saudi Arabia. Another reason could be more emphasis laid by the GPs offering vaccines for uptake. However, it is also important to note that COVID-19 vaccination was not mandatory in the UK and this would have had a positive effect on influenza vaccination uptake.

Influenza vaccine acceptance

The reported vaccine acceptance of the influenza vaccine among the overall study subjects after three years of the coronavirus pandemic in 2022 was 63.4%. Vaccine acceptance among those who were offered vaccines was 49.6%, and the reported vaccine hesitancy was 36.6%. Although not significant, women showed a higher level of acceptance. Higher vaccine acceptance was significantly associated with past episodes of seasonal influenza infection (p<0.001) and seasonal influenza vaccination history before the COVID-19 pandemic (p<0.001). Low acceptance of the influenza vaccine was observed during the COVID-19 pandemic (p<0.001). The present study showed some degree of consistency with other published studies from similar regions that showed a 31.7% willingness to receive the flu vaccine in 2021 [23]. On the contrary, our results differ from other published international studies. An Italian survey reported a 75% willingness to take the influenza vaccine during the pandemic year 2020-2021 [24]. Another study by Goldman et al. involving six countries demonstrated a 15.8% increase from the previous year of 54.2% willingness for the influenza vaccine after the COVID-19 pandemic [25].

The change in behavior was attributed to changes in risk perception due to the influence of the COVID-19 pandemic on the decision-making process. One main reason that could possibly justify the inconsistent findings of the present study with other international studies may be related to the mandatory vaccination of the three doses of COVID-19 vaccines in Saudi Arabia leading to lower acceptance of influenza vaccines. Another reason could be due to delayed peaks of seasonal influenza illness or a lower prevalence of seasonal influenza infections in Saudi Arabia during the first year of the COVID-19 pandemic due to stringent restrictions and lockdown measures to control the coronavirus pandemic. Evidence suggests that a combination of social distancing, mandatory mask-wearing, hand hygiene measures, restricted public movement and activities, online schooling, and limited onsite work reduced the local transmission of the influenza virus [26].

Factors associated with seasonal influenza vaccine acceptance

The driving factors responsible for acceptance of influenza vaccination were past history of influenza infection, previous immunization with influenza vaccine before and during the COVID-19 pandemic, and acceptance of influenza vaccine among the vaccine-offered group. No association was seen between influenza vaccine acceptance and smoking status, chronic illness, history of COVID-19 infection, or living with those susceptible to influenza sickness. Many studies have linked previous immunization practices as a strong predictor of influenza vaccine acceptance. Those subjects with a previous history of influenza vaccination were more likely to vaccinate against influenza in global as well as regional studies [15,24,25]. Therefore, the past behavior of immunization has been strongly linked to increased vaccine uptake. However, in the present study, the existing COVID-19 pandemic did not seem to positively influence the uptake of influenza vaccination, on the other hand, it had a detrimental effect.

Furthermore, another issue of concern to be highlighted is the low rate of vaccines offered by physicians. Only 28% of the study subjects reported having been offered vaccines. Vaccines offered by primary care physicians and behavioral changes in the subjects play a major role in stepping up uptake. Further studies are needed to understand the factors influencing vaccine acceptance behavior with a special focus related to the COVID-19 pandemic influence.

Knowledge related to influenza transmission and priority groups for vaccination

Two simple questions concerning the methods of transmission of the virus and who should be prioritized to receive the seasonal influenza vaccine were asked to assess the knowledge levels. Women seem to have more awareness related to influenza illness than men did. The highest priority for receiving the influenza vaccine was allotted to people living with healthcare workers, those having chronic comorbid conditions, and those aged more than 65 years, followed by children under 16 years. Pregnant women were the least chosen priority group for vaccination; however, a significant difference was observed in more women prioritizing than men (p=0.004).

The level of awareness of the transmission of the virus was markedly well both among male and female patients. Spread by cough and sneeze was rated as the most highly transmissible, followed by direct contact with an infected person and touching contaminated objects. The misleading question of eating infected meat leads to transmitting influenza sickness was well picked up by the majority and only 19.7% related it positively. Although the knowledge was higher, it did not reflect the behavior. In-depth studies are needed to determine the underlying reasons for lesser uptake.

Reasons for acceptance, denial, and sources of vaccine information

Vaccine acceptance and hesitancy depend on numerous factors. The three most-cited reasons for vaccine denial include an assumption of not being at risk of the disease, lack of information about the vaccine, and fear of possible side effects. These responses reflect the need for better awareness campaigns and elucidating information on the prevention of disease transmission and vaccine effectiveness. Furthermore, the most likely reasons for vaccine uptake include a decreased likelihood of getting influenza sickness for yourself and others, as well as vaccine safety. These factors guide the behavior and decision-making process for both vaccine denial and uptake. 

The Ministry of Health is a major source of information for learning about health and vaccines. Unlike in other countries where the primary source of health information is the GPs [24,27], in Saudi Arabia, the MOH plays a predominant role in generating health-related awareness information to the public. 

The study may contain certain limitations. The study population was limited to the patients attending the hospital, and hence the results may not be generalized to the entire population.

## Conclusions

In summary, the coronavirus pandemic has had certain repercussions and influenced the uptake of influenza vaccination in 2020 and 2021. The vaccine offer by the primary care physicians was found to be very low. However, once offered, participants' willingness to take the influenza vaccine improved. Caution must be exercised in the coming winter months of 2023, raising public awareness on increasing immunity and protection by vaccination for the influenza virus. Further studies are highly recommended to assess the impact of the pandemic on the uptake and acceptance of influenza vaccination on a larger population involving diverse groups. The physicians and the health officials must exercise greater efforts to increase the rate of annual influenza immunization.
